# A role for bronchial epithelial autotaxin in ventilator-induced lung injury

**DOI:** 10.1186/s40635-021-00379-7

**Published:** 2021-03-29

**Authors:** Ioanna Nikitopoulou, Ioanna Ninou, Nikolaos Manitsopoulos, Ioanna Dimopoulou, Stylianos E. Orfanos, Vassilis Aidinis, Anastasia Kotanidou

**Affiliations:** 1 1st Department of Critical Care Medicine & Pulmonary Services, GP Livanos and M Simou Laboratories, National and Kapodistrian University of Athens Medical School, Evangelismos Hospital, 45, Ipsilantou Street, Athens, Greece; 2 Institute of Immunology, Biomedical Sciences Research Center Alexander Fleming, Athens, Greece; 3 1st Department of Critical Care Medicine & Pulmonary Services, National and Kapodistrian University of Athens Medical School, Evangelismos Hospital, 45, Ipsilantou Street, Athens, Greece; 4 2nd Department of Critical Care, National and Kapodistrian University of Athens Medical School, Attikon” Hospital, Athens, Greece

**Keywords:** Mechanical ventilation, Autotaxin, Lung function, Edema

## Abstract

**Background:**

The pathophysiology of acute respiratory distress syndrome (ARDS) may eventually result in heterogeneous lung collapse and edema-flooded airways, predisposing the lung to progressive tissue damage known as ventilator-induced lung injury (VILI). Autotaxin (ATX; ENPP2), the enzyme largely responsible for extracellular lysophosphatidic acid (LPA) production, has been suggested to play a pathogenic role in, among others, pulmonary inflammation and fibrosis.

**Methods:**

C57BL/6 mice were subjected to low and high tidal volume mechanical ventilation using a small animal ventilator: respiratory mechanics were evaluated, and plasma and bronchoalveolar lavage fluid (BALF) samples were obtained. Total protein concentration was determined, and lung histopathology was further performed

**Results:**

Injurious ventilation resulted in increased BALF levels of ATX. Genetic deletion of ATX from bronchial epithelial cells attenuated VILI-induced pulmonary edema.

**Conclusion:**

ATX participates in VILI pathogenesis.

## Background

Mechanical ventilation strategies have been lifesaving in many patients suffering from acute respiratory distress syndrome (ARDS). ARDS may result in lung collapse and airspace flooding with protein-rich fluid due to alveolar capillary permeability [1]. ARDS accounts for 10% of intensive care unit (ICU) admissions, and 24% of patients receiving mechanical ventilation in the ICU [2]. Lung damage due to cyclic stretch caused by mechanical ventilation, in particular when the lung is injured or infected, is known as ventilator-induced lung injury (VILI). VILI can increase systemic inflammatory response, exacerbate pulmonary edema and contribute to the development of multi-οrgan failure and death.

ATX, originally identified as an autocrine motility-stimulating factor, is a secreted lysophospholipase D (lysoPLD), largely responsible for the synthesis of extracellular lysophosphatidic acid (LPA) through the hydrolysis of lysophosphatidylcholine (LPC) [3]. ATX is expressed by various cell types and tissues, including the bronchial epithelial cells of the lung from where it is constitutively secreted in the bronchoalveolar lavage fluid (BALF) [4, 5]. The enzymatic product of ATX, LPA, is a growth factor-like phospholipid that evokes multiple effects in almost all cell types through its G protein-coupled receptors (GPCRs; LPAR1-6), which show widespread distribution and participate in signal transduction pathways [6]. In the lung, LPA has been reported to modulate various properties of pulmonary cells, including endothelial permeability, epithelial apoptosis, as well as fibroblast and smooth muscle homeostasis [7, 8].

A pathologic role for ATX has been suggested in different chronic inflammatory diseases and cancer [5, 9]. In the lung, where ATX has been identified as a candidate gene involved in the control of pulmonary functions [10], increased ATX and/or LPA expression has been reported in asthma and chronic obstructive pulmonary disease, as well as in idiopathic pulmonary fibrosis (IPF) [8]. Genetic or pharmacologic ATX silencing prevented the development of bleomycin-induced pulmonary fibrosis [4, 11], leading to ongoing phase III clinical trials in IPF patients [12]. Vascular leak has been suggested to be among the dominant pathological effects of ATX/LPA in pulmonary pathophysiology and fibrosis [8, 13], a dysfunction characterizing VILI pathogenesis [14]. Therefore, in this study we aimed to investigate a possible role for ATX in lung injury caused by mechanical ventilation.

## Methods

### Mice

Mice were bred at the animal facilities of Biomedical Sciences Research Center ‘Alexander Fleming’, under specific pathogen-free conditions, at 20–22 °C, 55 ± 5% humidity, and a 12-h light–dark cycle; food and water were provided ad libitum. Mice were bred and maintained in a C57BL/6 genetic background for more than 10 generations. All experimentation was approved by the Institutional Animal Ethical Committee (IAEC) of Biomedical Sciences Research Center “Alexander Fleming”, as well as by the Veterinary Service of the governmental prefecture of Attica, Greece (approval protocol number K/1889/2011). The study was conducted in compliance with the European Union Directive 2010/63/EU on animal experimentation. All efforts were made to minimize animal distress and suffering. The health status of mice was daily monitored. Mice were euthanized under deep anesthesia by exsanguination. The generation and genotyping instructions of ATX^n/n^ conditional knockout mice [15] and CC10-Cre [4] mice have been previously described.

### Ventilator-induced lung injury (VILI)

Mouse trachea was exposed under sterile conditions, while animals were under deep anesthesia by ketamine/xylazine (100 and 10 mg/Kg, respectively). A 22-gauge catheter was used for the cannulation of the trachea. Mechanical ventilation was performed using a small animal ventilator (FlexiVent, Scireq, Ontario, Canada). Tidal volume (TV) was set at 8 mL/kg while positive end-expiratory pressure (PEEP) was set at 2 cmH_2_O. A brief run-in period was followed by two deep inflations to total lung capacity for standardization purposes. After 1 min of low tidal volume ventilation, baseline measurements of lung function were obtained. Then, low or high tidal volume ventilation was performed according to experimental design.

### Respiratory mechanics

Respiratory mechanics were evaluated by measuring tissue elastance coefficient (H) and tissue damping coefficient (G) via forced-oscillation technique. This is achieved by the use of the Constant Phase model. The successive measurements were obtained in 30-s intervals [16]. Quasi-static pressure–volume curves were transduced in order to measure the static compliance (Cst) of the respiratory system by fitting the Salazar–Knowles equation to the expiratory branch of the PV loop [17]

### Samples isolation

Venous blood samples were obtained from the abdominal inferior vena cava via a heparinized 27g syringe. Plasma samples were obtained via centrifugation of the blood samples at 1500 rpm for 10 min at 4 °C and stored at − 80 °C. Broncho alveolar lavage fluid (BALF) was obtained via tracheotomy by injecting and slowly withdrawing 1 ml of phosphate-buffered saline (PBS). After repeating this procedure 3 times, the fluid was separated from cellular components by centrifugation at 1500 rpm for 10 min at 4 °C, and supernatants were stored at − 80 °C. The lungs were exposed by a mid-thoracotomy incision; the right lung was frozen in liquid nitrogen and stored at − 80 °C for further analysis. The left lung was inflated with 4% neutral buffered paraformaldehyde; the trachea was tied, and the left lung was immersed in 4% neutral buffered paraformaldehyde for 24 h before embedding in paraffin.

### BALF total protein and total cell count

Total protein concentration in the BALF was determined with the Bio-Rad Dc Protein Assay kit (Bio-Rad Laboratories, Hercules, CA, USA) according to the manufacturer's instructions. Total cell counting in BALF was performed manually using an improved Neubauer hemocytometer according to common procedures.

### ATX ELISA

Samples and standards were diluted in coating buffer (12 mM NaCO_3_ and 28 mM NaHCO_3_, pH 9.6) and incubated overnight at 4 °C. Recombinant ATX (C187-EN, R&D Systems) at concentrations 120–3.75 ng/ml was used to construct a linear standard curve. After blocking with 2% BSA in PBS–Tween, samples were incubated for 2 h with anti-ATX goat anti-mouse antibody. The anti-ATX antibody was detected with horse anti-goat HRP conjugated antibody (PI-9500, Vector, UK) and developed with TMB (3,3′,5,5′-tetramethylbenzidine, Sigma). The reaction was stopped with 2 M H_2_SO_4_ and readings were obtained using a spectrophotometer at 450 nm.

### ATX activity assay

ATX/LysoPLD activity was measured using the TOOS activity assay. Hydrogen peroxide is used as the oxidizing agent and in the presence of horseradish peroxidase, it reacts with TOOS (N-ethyl-N-(2-hydroxy-3-sulfopropyl)-3-methylaniline) and 4-AAP (aminoantipyrene) to form a pink quinoneimine dye which absorbs at 555 nm. 1 × LysoPLD buffer (100 mM Tris–HCl pH 9.0, 500 mM NaCl, 5 mM MgCl_2_, 5 mM CaCl_2_, 60 μM CoCl_2_, 1 mM LPC) was incubated at 37 °C for 30 min. Plasma and BALF samples were incubated with 1 × LysoPLD buffer at 37 °C for 4 h. At the end of the incubation, a color mix (0.5 mM 4-AAP, 7.95 U/ml HRP, 0.3 mM TOOS, 2 U/ml choline oxidase in 5 mM MgCl_2_/50 mM Tris–HCl pH 8.0) was prepared and added to each well. Absorbance (A) was measured at 555 nm every 5 min for 20 min. For each sample, the absorbance was plotted against time and the slope (dA/min) was calculated for the linear portion of each reaction. ATX activity was calculated according to the following equation: activity (U/ml) = (μmol/min/ml) = [dA/ min(sample) – dA/min(blank)] × Vt/(e×Vs × 0.5) where *Vt* total volume of reaction (ml), *Vs* volume of sample (ml), e: millimolar extinction coefficient of quinoneimine dye under the assay conditions (e = 32, 8 μmol/cm^2^) and 0.5: the moles of quinoneimine dye produced by 1 mol of H_2_O_2_.

### H&E staining

5-μm sections of paraffin-embedded lungs were stained with hematoxylin and eosin according to standard histological procedures. Microscopic lung structure was observed under an Olympus (BX50F4) microscope.

### Statistical analysis

Data are presented as medians with interquartile range. Pairwise comparisons among groups were assessed using the Mann–Whitney test. Multiple comparisons were assessed by the Kruskal–Wallis test. Statistical analyses were conducted with the statistical program Graph Pad Prism vol. 5 (Graph Pad Software, Inc. CA USA). Statistical differences are considered significant when p < 0.05 (significant differences shown by *).

## Results

### Increased pulmonary ATX levels upon ventilation-induced lung injury (VILI)

**Fig. 1:**
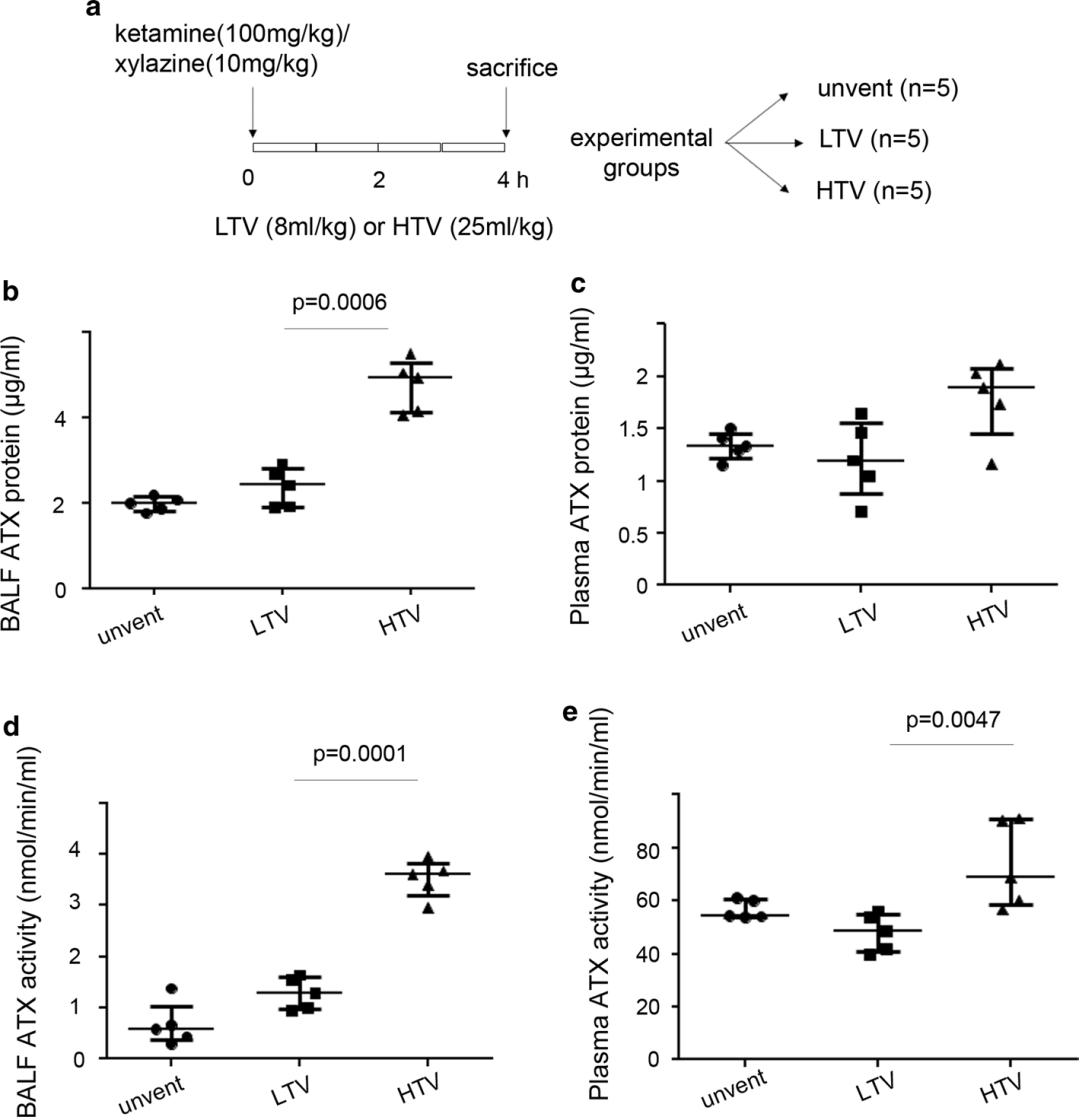
Increased ATX concentrations in the BALFs and plasma of ventilated mice. **a** Graphic representation of the experimental groups studied. **b** BALF ATX concentrations were measured using an ELISA assay. **c** ATX concentrations in plasma were determined by ELISA. **d**, **e** ATX activity in BALFs and in plasma, respectively, was measured using a modified Amplex Red PLD activity kit. *n* = 5 mice per group. Data are expressed as medians with interquartile range (*statistically significant differences, *P* < 0.05) *LTV* low tidal volume, *HTV* high tidal volume, *ATX* autotaxin, *BALF* bronchoalveolar lavage fluid, *PLD* phospholipase D

To examine a possible involvement of ATX in the pathogenetic mechanisms underlying VILI, we first sought to measure ATX protein and activity levels in plasma and BALF upon VILI. To that end, wild-type C57Bl6/J mice were randomly assigned to 3 groups (*n* = 5/group; Fig. 1a) and connected to a small animal ventilator, as described in Methods. The first group was not subjected to ventilation (unvent); the second was ventilated at low tidal volume for 4 h (LTV; 8 mL/kg) and the third group was ventilated at high tidal volume for 4 h (HTV; 25 mL/kg) (Fig. 1a).

High-volume ventilation and the associated lung injury was found to increase ATX protein levels in the BALFs (Fig. 1b), but not statistically significantly in the plasma (Fig. 1c). To possibly correlate ATX protein and its activity levels, samples were then processed in order to determine ATX enzymatic activity with the TOOS assay, where the lysophosphatidylcholine substrate is cleaved by ATX, liberating LPA and choline. Choline is then oxidized by choline oxidase giving hydrogen peroxide which in turn reacts with TOOS (N-ethyl-N-(2-hydroxy-3-sulfopropyl)-3-methylaniline). ATX activity, in agreement with the protein levels, was found significantly increased in BALF samples upon high tidal volume ventilation (Fig. 1d); a much lower increase was detected in plasma (Fig. 1e). Therefore, VILI induces local pulmonary ATX expression and activity.

### Bronchial epithelial cell-derived ATX participates in VILI pathogenesis

**Fig. 2:**
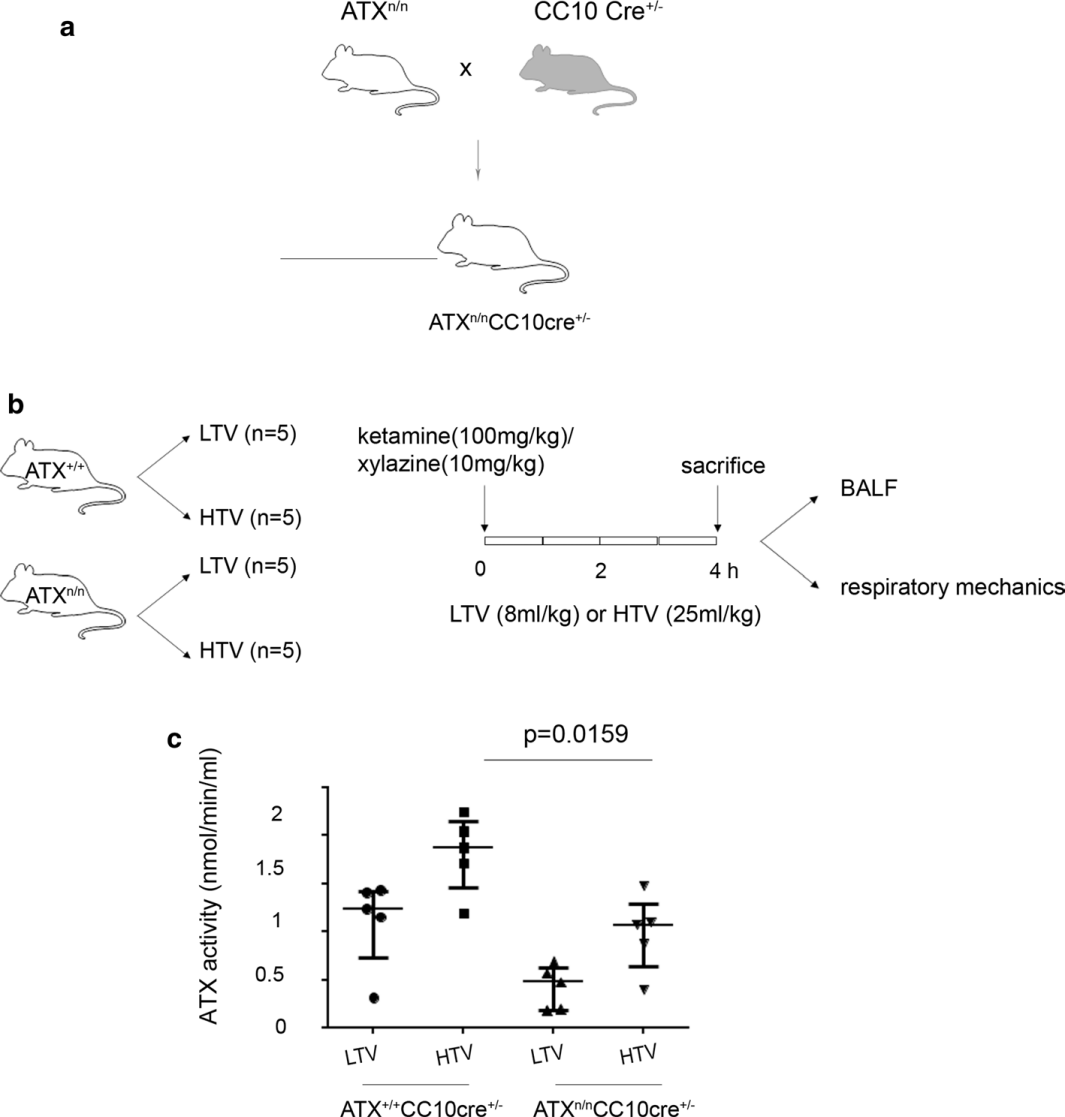
Tissue-specific deletion of ATX from bronchial epithelial cells reduces BALF activity. **a** Schematic representation of mating strategy for ATX deletion from Clara cells. **b** Graphic representation of the experimental groups studied. **c** Total ATX activity levels in BALF of ATX^n/n^CC10Cre^+/-^ mice were found downregulated with the TOOS assay. Data are expressed as medians with interquartile range, *n* = 5 per group. *ATX* autotaxin, *CC10* Clara cell 10-kDa protein, *LTV* low tidal volume, *HTV* high tidal volume, *BALF* bronchoalveolar lavage fluid

Given the increased ATX levels upon mechanical ventilation, and to examine the possible pathogenic role of ATX in VILI, ATX was then conditionally ablated specifically in bronchial epithelial cells, through the mating of the ATX^n/n^ mouse with the transgenic Clara cell 10-kDa protein (CC10)-Cre murine strain (Fig. 2a). CC10-Cre drives ATX recombination specifically in bronchial epithelial cells with an efficiency of 70–80% [4]. Genetically modified mice were then subjected to low or high tidal volume ventilation (LTV and HTV, respectively) together with their littermate controls (Fig. 2b). Genetic deletion of ATX from bronchial epithelial cells resulted in reduced ATX activity levels in the BALF of ATX^n/n^CC10Cre^+/-^ mice (Fig. 2c), reconfirming ATX expression from bronchial epithelial cells and the efficiency of the conditional genetic deletion.

**Fig. 3:**
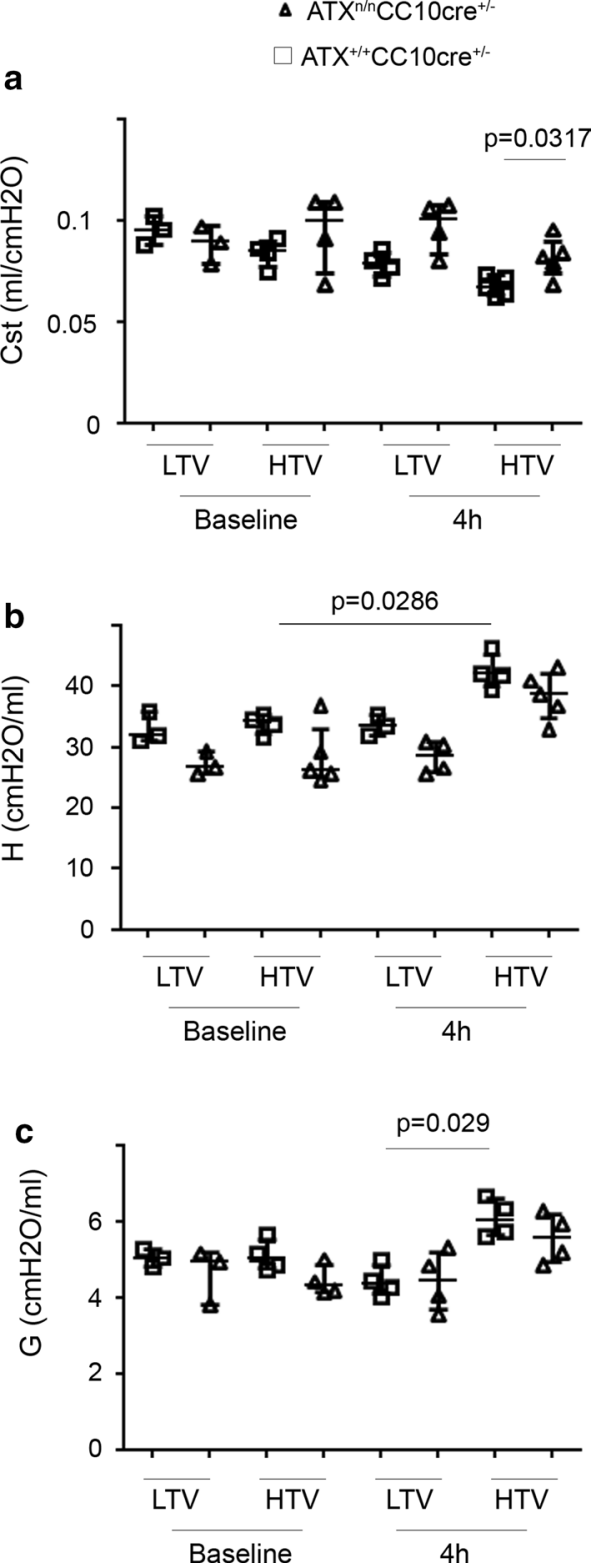
Evaluation of lung mechanics in all animal groups upon ventilator injury. Lung function parameters were measured using the flexiVent system in mice undergoing ventilation with high (25 ml/kg) or low (8 ml/kg) tidal volume for 4 h. **a** Static compliance was evaluated by a single quasi-static pressure–volume curve. Lung tissue elastance **b** and lung tissue resistance **c** were evaluated via forced-oscillation technique (*n* = 5 per group). *ATX* autotaxin, *CC10* Clara cell 10-kDa protein, *LTV* low tidal volume, *HTV* high tidal volume, *Cst* static compliance, *H* lung tissue elastance, *G* lung tissue damping

Alterations in respiratory mechanics were observed at the 4-h time point (Fig. 3). As expected, ventilation with high tidal volume led to impaired lung mechanics. More specifically, quasi-static respiratory compliance (Cst) was measured via pressure–volume curves and was decreased upon VILI (Fig. 3a). Lung tissue elastance coefficient (H) and tissue damping coefficient (G) were measured via the forced-oscillation technique and showed the expected increase following injurious ventilation (Fig. 3b, c). These responses to injurious mechanical ventilation seemed to be slightly affected in mice lacking ATX from bronchiolar epithelial cells. In particular, compliance upon high tidal volume was reduced in mice lacking ATX in bronchial epithelial cells (ATX^n/n^CC10Cre^+/-^), compared to control mice (Fig. 3a). As for elastance values, these peaked at 42.7 ± 1.95 cmH_2_O/ml in ATX^+/+^CC10Cre^±^ mice after 4 h of HTV ventilation (Fig. 3b). Respective values in ATX^n/n^CC10Cre^+/-^ mice (38.9 ± 2.18 cmH_2_O/ml), indicated less impaired lung function. Finally, tissue-specific ATX deletion seemed to partially affect tissue damping values, as they were found decreased in mice lacking bronchial epithelial ATX, although not in a statistically significant way (Fig. 3c).

**Fig. 4:**
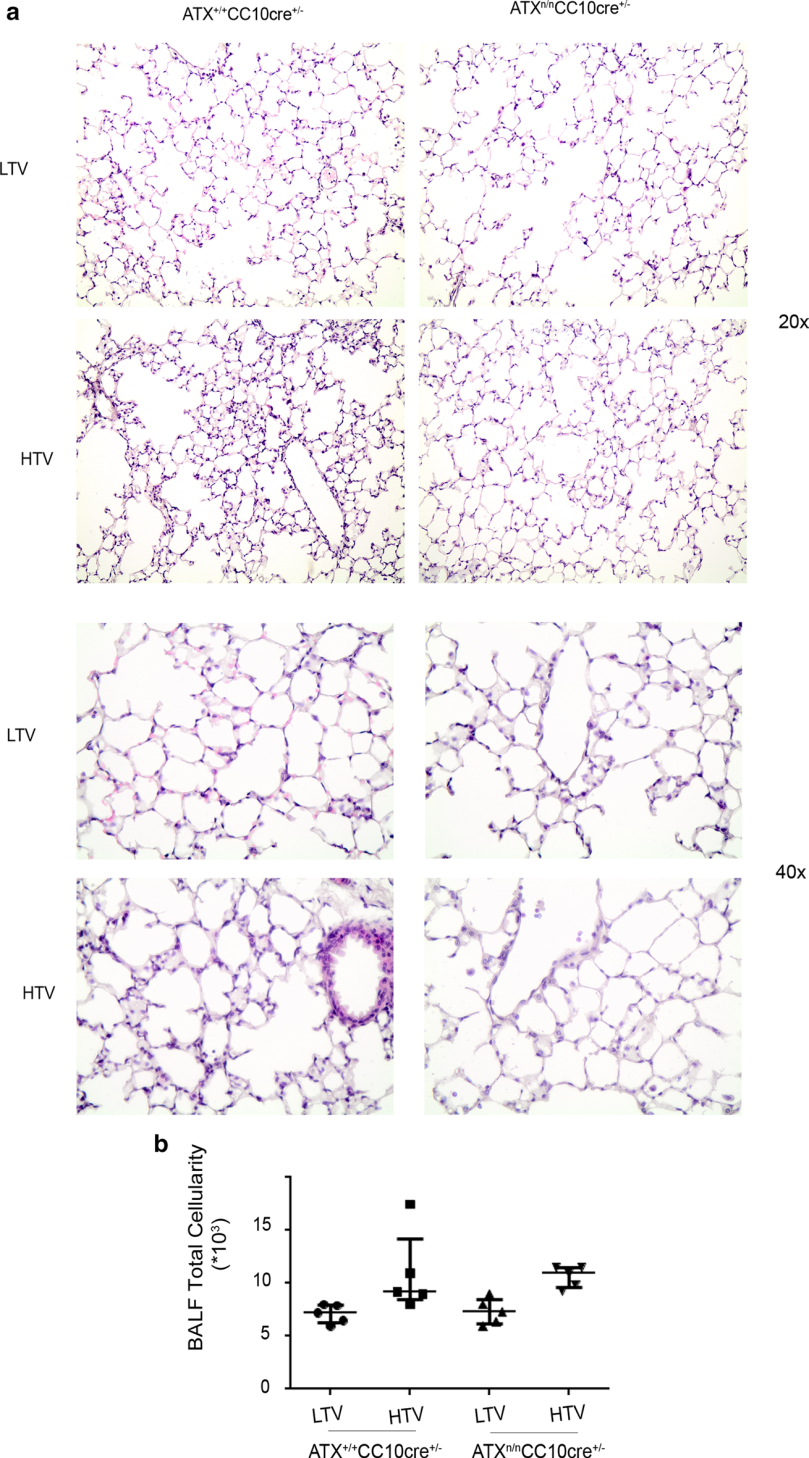
Deletion of ATX from bronchial epithelial cells improves lung histopathology. **a** Representative hematoxylin and eosin-stained lung sections at 20 × and 40 × magnification. **b** Total cell counts in BALFs. *Statistically significant differences (*P* < 0.05), *n* = 5 per group. *ATX* autotaxin, *CC10* Clara cell 10-kDa protein, *LTV* low tidal volume, *HTV* high tidal volume, *BALF* bronchoalveolar lavage fluid

To further examine the possible involvement of ATX expression from bronchial epithelial cells in lung injury caused by mechanical ventilation, the pulmonary inflammatory response upon HTV was assessed in the aforementioned experimental groups. Conditional genetic deletion of ATX from bronchial epithelium ameliorated lung architecture upon injurious ventilation (Fig. 4). More specifically ATX^n/n^CC10Cre^+/-^ mice ventilated at HTV of 25 mL/kg for 4 h showed an improved lung histopathology, recognizable as less edema formation and interstitial cell infiltration. The hematoxylin and eosin staining results showed that the alveolar morphology of mice with conditionally ablated ATX (ATX^n/n^CC10Cre^+/-^) lacked inflammatory cell infiltration. In littermate controls (ATX^+/+^CC10Cre^+/-^), HTV resulted in disordered alveolar structure, with thickening of the alveolar space and lung infiltration of inflammatory cells. To further assess the inflammatory response to mechanical ventilation, total cell counts in BALF were determined, a marker of general pulmonary inflammation. Inflammatory BALF cells were found reduced in the absence of ATX, albeit not statistically significantly, given the relatively small group size (Fig. 4b).

**Fig. 5:**
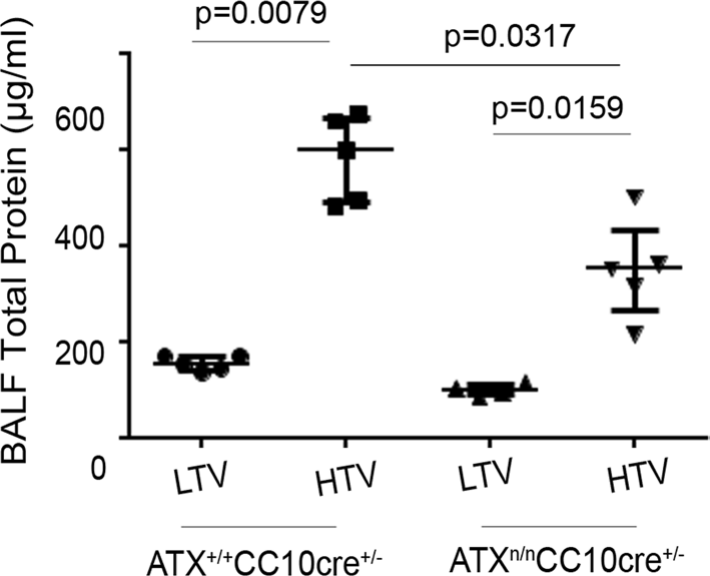
Deletion of ATX from bronchial epithelial cells reduces edema and inflammation due to injurious ventilation. Total protein concentrations in BALFs of mice with (ATX^+/+^CC10Cre^+/-^) and without (ATX^n/n^CC10Cre^+/-^) ATX expression in bronchial epithelium. *ATX* autotaxin, *CC10* Clara cell 10-kDa protein, *LTV* low tidal volume, *HTV* high tidal volume, *BALF* bronchoalveolar lavage fluid

Endothelial barrier disruption represents a major feature of acute lung injury, leading to flooding of pulmonary air space with protein-rich fluid. Therefore, we then examined endothelial permeability and pulmonary edema upon VILI, via the quantification of total protein levels in BALF, as commonly used [18, 19] and as previously reported [16]; total protein levels have been shown to well-correlate with wet-to-dry lung ratios [20], a traditional way of quantifying pulmonary edema. The CC10 genetic deletion of ATX and the attenuation of local ATX levels in the lung significantly affected BALF total protein levels. More specifically, protein leakage into the alveolar space was found lower in ATX^n/n^CC10Cre^+/-^ mice compared to ATX^+/+^CC10Cre^+/-^ littermate controls (Fig. 5), indicating attenuation of VILI-induced barrier dysfunction upon ATX tissue-specific deletion.

## Discussion

Growing evidence supports a major role for ATX, the enzyme responsible for the production of extracellular LPA, in inflammatory conditions, cancer and pulmonary fibrosis [5, 8], rendering it a promising therapeutic target. Yet, possible involvement of ATX in VILI pathophysiology remained unknown. In this study, we observed for the first time that ATX is significantly upregulated in plasma the BALF of mice subjected to high tidal volume ventilation (HTV; 25 mL/kg, 4 h). Bronchial epithelium-specific deletion of ATX reduced the severity of pulmonary edema, suggesting a role for ATX in the pathogenesis of the disease.

ATX exerts most of its effects through the extracellular production of LPA, a bioactive phospholipid which in turn signals through six different receptors [6, 21]. ATX has been shown to have a broad tissue distribution, with the highest concentrations in the brain, ovary, placenta, kidney, intestine, and lung [22]. Particularly for the lung, ATX mRNA expression has been previously localized to normal bronchial epithelium by in situ hybridization [23]. As previously reported, conditional ATX recombination in ATX^n/n^CC10Cre^+/-^ mice occurs in bronchial epithelial cells, with no effects on lung architecture due to the transgenic Cre driver mouse strain itself [4].

Deletion of ATX from bronchial epithelial cells had a minor effect in preserving lung function. Lung mechanics were measured using the forced-oscillation technique, which yields data on large airways resistance, on tissue elastance and resistance that reflects the function of the peripheral parts of the lung [24]. As shown in this study, mechanical derangements due to high ventilation were only partially attenuated by ATX deletion from bronchial epithelial cells. Differences between static compliance values, but not between lung elastance and resistance were significant. Similarly, deletion of ATX from bronchial epithelium was previously shown to have minor effect on endotoxin-induced acute lung injury development [25]. These observed effects in VILI pathology disagree with the proven role of ATX in the development of bleomycin-induced chronic pulmonary inflammation and fibrosis [2], suggesting a differential involvement of the ATX/LPA axis in acute versus chronic inflammation. Notably, in lung tissues from IPF patients ATX was found to localize mainly in the bronchial epithelium and a similar ATX staining profile was observed in the lungs of mice treated with bleomycin (BLM) [2], while increased ATX levels were detected in the corresponding BALFs [26]. Systemic pharmacologic inhibition of ATX managed to attenuate pulmonary fibrosis [9, 27] and to prevent BLM-induced pulmonary fibrosis [28]. A distinctive implication of ATX in acute and chronic inflammation is thus proposed; in acute conditions effects are exerted to a smaller extent than in chronic and possibly require a long-term exposure. The distinctive roles could be also possibly related to ATX expression by alveolar macrophages [9]. Several studies have suggested that alveolar macrophages are involved in the pathogenesis of VILI and participate in the inflammatory response [29]. Specifically, mechanical stretch stimulation can activate macrophages, which can then recruit peripheral neutrophils to the lungs [30].

In our model, ventilation of mice with 25 mL/kg leads to microvascular barrier failure, documented by protein-enriched fluid accumulation. We report here that deletion of ATX from bronchial epithelial cells managed to reduce pulmonary edema induced by HTV ventilation, represented by total protein levels in BALF, a well-accepted marker of endothelial permeability and pulmonary edema [31]. Reduction of edema in our mouse model is further reflected in the lung histopathology, as mice lacking ATX in bronchial epithelium show less alveolar wall thickening upon HTV ventilation.

Permeability edema has been previously connected with loosening of inter-endothelial adhesion structures [32], and LPA is a molecule known to regulate vascular homeostasis [33, 34]. The effect of LPA signaling in vascular development, was previously shown by the vascular defects participating in the embryonic lethal phenotype of ATX knockout mice [15]. Excess ATX–LPA signaling also induces severe vascular defects, indicating that excess LPA can inhibit angiogenesis and thus should be regulated [35]. In adult life, LPA has been suggested to regulate endothelial cell physiology, through signaling pathways controlling the transcription of angiogenic genes [34]. LPA can also promote endothelial permeability, as shown by the attenuation of BLM-induced vascular leak in LPA receptor 1 knockout mice [8]. Therefore, the proven effects of LPA on endothelial cell migration, proliferation and barrier stability could explain edema reduction in ATX^n/n^CC10Cre^+/-^ mice [36].

### Study limitations

An animal model recapitulates some of the features of a human disease, but not all, and results are not always translatable in the human disease. In this context, and although the model of VILI used here is widely accepted and utilized, further clinical studies will be required to establish a human relevance. However, preliminary results (data not shown) suggest an increase in the serum ATX levels upon mechanical ventilation of COVID19 patients. Concerning the model per se, as utilized here, the small group size, as well as the number and nature of readout assays, limit the depth of the conclusions; however, the model is very reproducible, as it depends on automated mechanical pathogenic stimuli, resulting in readout assays that well-correlate to each other. Moreover, and most importantly, the genetic studies presented here need to be complemented with pharmacologic studies with an ATX inhibitor. As ATX has emerged as a therapeutic target in idiopathic pulmonary fibrosis (IPF) [12], many novel compounds are increasingly reported [37] and will be likely soon commercially available, thus allowing pharmacologic studies in vivo.

## Conclusions

Ventilator-induced lung injury entails an upregulation of ATX protein and activity levels in the BALFs, suggesting a possible involvement of ATX and associated LPA signaling in VILI pathogenesis. Accordingly, conditional genetic deletion of ATX from bronchial epithelial cells and the attenuation of its levels in the BALFs, reduced vascular leak upon VILI, reconfirming bronchial epithelial secretion of ATX and establishing a pathogenic role of ATX in VILI, pending pharmacologic and clinical studies.

## Data Availability

The datasets analyzed during the current study are available from the corresponding author on reasonable request.

## Abbreviations

ARDS: Acute respiratory distress syndrome
ICU: Intensive care unit
VILI: Ventilator-induced lung injury
ATX: Autotaxin
LPA: Lysophosphatidic acid
LPC: Lysophosphatidylcholine
GPCR G: Protein-coupled receptor
PLD: Phospholipase D
BALF: Bronchoalveolar lavage fluid
PEEP: Positive end-expiratory pressure
TV: Tidal volume
IPF: Idiopathic pulmonary fibrosis
BLM: Bleomycin

## References

[1] Nieman GF, Andrews P, Satalin J, Wilcox K, Kollisch-Singule M, Madden M, Aiash H, Blair SJ, Gatto LA, Habashi NM (2018). Acute lung injury: how to stabilize a broken lung. Crit Care (London, England).

[2] Fan E, Brodie D, Slutsky AS (2018). Acute Respiratory Distress Syndrome: Advances in Diagnosis and Treatment. JAMA.

[3] Umezu-Goto M, Kishi Y, Taira A, Hama K, Dohmae N, Takio K, Yamori T, Mills GB, Inoue K, Aoki J (2002). Autotaxin has lysophospholipase D activity leading to tumor cell growth and motility by lysophosphatidic acid production. J Cell Biol.

[4] Oikonomou N, Mouratis MA, Tzouvelekis A, Kaffe E, Valavanis C, Vilaras G, Karameris A, Prestwich GD, Bouros D, Aidinis V (2012). Pulmonary autotaxin expression contributes to the pathogenesis of pulmonary fibrosis. Am J Respir Cell Mol Biol.

[5] Magkrioti C, Galaris A, Kanellopoulou P, Stylianaki EA, Kaffe E, Aidinis V (2019). Autotaxin and chronic inflammatory diseases. J Autoimmun.

[6] Yung YC, Stoddard NC, Chun J (2014). LPA receptor signaling: pharmacology, physiology, and pathophysiology. J Lipid Res.

[7] Magkrioti C, Aidinis V (2013). ATX and LPA signalling in lung pathophysiology. World J Respirol.

[8] Ninou I, Magkrioti C, Aidinis V (2018). Autotaxin in Pathophysiology and Pulmonary Fibrosis. Frontiers in medicine.

[9] Barbayianni E, Kaffe E, Aidinis V, Kokotos G (2015). Autotaxin, a secreted lysophospholipase D, as a promising therapeutic target in chronic inflammation and cancer. Prog Lipid Res.

[10] Ganguly K, Stoeger T, Wesselkamper SC, Reinhard C, Sartor MA, Medvedovic M, Tomlinson CR, Bolle I, Mason JM, Leikauf GD (2007). Candidate genes controlling pulmonary function in mice: transcript profiling and predicted protein structure. Physiol Genomics.

[11] Ninou I, Kaffe E, Muller S, Budd DC, Stevenson CS, Ullmer C, Aidinis V (2018). Pharmacologic targeting of the ATX/LPA axis attenuates bleomycin-induced pulmonary fibrosis. Pulm Pharmacol Ther.

[12] Maher TM, Kreuter M, Lederer DJ, Brown KK, Wuyts W, Verbruggen N, Stutvoet S, Fieuw A, Ford P, Abi-Saab W (2019). Rationale, design and objectives of two phase III, randomised, placebo-controlled studies of GLPG1690, a novel autotaxin inhibitor, in idiopathic pulmonary fibrosis (ISABELA 1 and 2). BMJ Open Resp Res.

[13] Tager AM, LaCamera P, Shea BS, Campanella GS, Selman M, Zhao Z, Polosukhin V, Wain J, Karimi-Shah BA, Kim ND (2008). The lysophosphatidic acid receptor LPA1 links pulmonary fibrosis to lung injury by mediating fibroblast recruitment and vascular leak. Nat Med.

[14] Dreyfuss D, Saumon G (1998). Ventilator-induced lung injury: lessons from experimental studies. Am J Respir Crit Care Med.

[15] Fotopoulou S, Oikonomou N, Grigorieva E, Nikitopoulou I, Paparountas T, Thanassopoulou A, Zhao Z, Xu Y, Kontoyiannis DL, Remboutsika E (2010). ATX expression and LPA signalling are vital for the development of the nervous system. Dev Biol.

[16] Manitsopoulos N, Orfanos SE, Kotanidou A, Nikitopoulou I, Siempos I, Magkou C, Dimopoulou I, Zakynthinos SG, Armaganidis A, Maniatis NA (2015). Inhibition of HMGCoA reductase by simvastatin protects mice from injurious mechanical ventilation. Respir Res.

[17] Manali ED, Moschos C, Triantafillidou C, Kotanidou A, Psallidas I, Karabela SP, Roussos C, Papiris S, Armaganidis A, Stathopoulos GT (2011). Static and dynamic mechanics of the murine lung after intratracheal bleomycin. BMC Pulm Med.

[18] Chimenti L, Luque T, Bonsignore MR, Ramírez J, Navajas D, Farré R (2012). Pre-treatment with mesenchymal stem cells reduces ventilator-induced lung injury. Eur Respir J.

[19] Ko YA, Yang MC, Huang HT, Hsu CM, Chen LW (2013). NF-κB activation in myeloid cells mediates ventilator-induced lung injury. Respir Res.

[20] Parker JC, Townsley MI (2004). Evaluation of lung injury in rats and mice. Am J Physiol Lung Cell Mol Physiol.

[21] Aikawa S, Hashimoto T, Kano K, Aoki J (2015). Lysophosphatidic acid as a lipid mediator with multiple biological actions. J Biochem.

[22] Giganti A, Rodriguez M, Fould B, Moulharat N, Coge F, Chomarat P, Galizzi JP, Valet P, Saulnier-Blache JS, Boutin JA (2008). Murine and human autotaxin alpha, beta, and gamma isoforms: gene organization, tissue distribution, and biochemical characterization. J Biol Chem.

[23] Yang Y, Mou L, Liu N, Tsao MS (1999). Autotaxin expression in non-small-cell lung cancer. Am J Respir Cell Mol Biol.

[24] Bates JH, Rincon M, Irvin CG (2009). Animal models of asthma. Am J Physiol Lung Cell Mol Physiol.

[25] Mouratis MA, Magkrioti C, Oikonomou N, Katsifa A, Prestwich GD, Kaffe E, Aidinis V (2015). Autotaxin and endotoxin-induced acute lung injury. PLoS ONE.

[26] Black KE, Berdyshev E, Bain G, Castelino FV, Shea BS, Probst CK, Fontaine BA, Bronova I, Goulet L, Lagares D (2016). Autotaxin activity increases locally following lung injury, but is not required for pulmonary lysophosphatidic acid production or fibrosis. FASEB J.

[27] Desroy N, Housseman C, Bock X, Joncour A, Bienvenu N, Cherel L, Labeguere V, Rondet E, Peixoto C, Grassot JM (2017). Discovery of 2-[[2-Ethyl-6-[4-[2-(3-hydroxyazetidin-1-yl)-2-oxoethyl]piperazin-1-yl]-8-methyli midazo[1,2-a]pyridin-3-yl]methylamino]-4-(4-fluorophenyl)thiazole-5-carbonitrile (GLPG1690), a first-in-class autotaxin inhibitor undergoing clinical evaluation for the treatment of idiopathic pulmonary fibrosis. J Med Chem.

[28] Nikolaou A, Ninou I, Kokotou MG, Kaffe E, Afantitis A, Aidinis V, Kokotos G (2018). Hydroxamic acids constitute a novel class of autotaxin inhibitors that exhibit in vivo efficacy in a pulmonary fibrosis model. J Med Chem.

[29] Dai H, Pan L, Lin F, Ge W, Li W, He S (2015). Mechanical ventilation modulates Toll-like receptors 2, 4, and 9 on alveolar macrophages in a ventilator-induced lung injury model. J Thorac Dis.

[30] Jaecklin T, Otulakowski G, Kavanagh BP (2010). Do soluble mediators cause ventilator-induced lung injury and multi-organ failure?. Intensive Care Med.

[31] Matute-Bello G, Downey G, Moore BB, Groshong SD, Matthay MA, Slutsky AS, Kuebler WM (2011). An official American Thoracic Society workshop report: features and measurements of experimental acute lung injury in animals. Am J Respir Cell Mol Biol.

[32] Vestweber D (2000). Molecular mechanisms that control endothelial cell contacts. J Pathol.

[33] Kazlauskas A (2015). Lysophosphatidic acid contributes to angiogenic homeostasis. Exp Cell Res.

[34] Chen Y, Ramakrishnan DP, Ren B (2013). Regulation of angiogenesis by phospholipid lysophosphatidic acid. Front Biosci (Landmark edition).

[35] Yukiura H, Kano K, Kise R, Inoue A, Aoki J (2015). Autotaxin overexpression causes embryonic lethality and vascular defects. PLoS ONE.

[36] Ren H, Panchatcharam M, Mueller P, Escalante-Alcalde D, Morris AJ, Smyth SS (2013). Lipid phosphate phosphatase (LPP3) and vascular development. Biochem Biophys Acta.

[37] Matralis AN, Afantitis A, Aidinis V (2019). Development and therapeutic potential of autotaxin small molecule inhibitors: from bench to advanced clinical trials. Med Res Rev.

